# Secondhand smoke exposure and risk of wheeze in early childhood: a prospective pregnancy birth cohort study

**DOI:** 10.1186/s12971-017-0138-7

**Published:** 2017-07-18

**Authors:** Keiko Tanaka, Yoshihiro Miyake, Shinya Furukawa, Masashi Arakawa

**Affiliations:** 1 Department of Epidemiology and Preventive Medicine, Ehime University Graduate School of Medicine, Shitsukawa, Toon, Ehime, 791-0295 Japan; 2 Epidemiology and Medical Statistics Unit, Translational Research Center, Ehime University Hospital, Ehime, Japan; 3 Health Tourism Research Fields, Graduate School of Tourism Sciences, University of the Ryukyus, Okinawa, Japan

**Keywords:** Child, Japan, Prospective studies, Secondhand smoke exposure, Wheeze

## Abstract

**Background:**

Evidence regarding the independent and additive effects of both pre- and postnatal smoking exposure on the risk of wheeze in children is limited. The purpose of this prospective pregnancy birth cohort study was to examine the association between prenatal and postnatal tobacco smoke exposure during the first year of life and the risk of wheeze in Japanese children aged 23 to 29 months.

**Methods:**

Study subjects were 1354 Japanese mother-child pairs. Information on the variables under study was obtained using repeated questionnaires that were completed by mothers, first prior to delivery, then shortly after birth and subsequently around 4, 12, and 24 months after delivery. Wheeze was defined according to the criteria of the International Study of Asthma and Allergies in Childhood.

**Results:**

Compared with no maternal smoking during pregnancy, maternal smoking throughout pregnancy was significantly associated with an increased risk of wheeze in children, yet there were no associations between maternal smoking in the first trimester only or in the second and/or third trimesters and the risk of wheeze. No association was observed between postnatally living with at least one household smoker and the risk of wheeze. An analysis to assess the additive effect of prenatal and postnatal smoking exposure revealed that, compared with children not exposed to maternal smoking during pregnancy and not postnatally living with at least one household smoker, those who were both exposed to maternal smoking during pregnancy and postnatally living with at least one household smoker had twofold odds of developing wheeze.

**Conclusions:**

Our findings suggest that maternal smoking throughout pregnancy might be associated with an increased risk of wheeze in children. There is also the possibility of a positive additive effect of pre- and postnatal smoking exposure on the risk of childhood wheeze.

**Electronic supplementary material:**

The online version of this article (doi:10.1186/s12971-017-0138-7) contains supplementary material, which is available to authorized users.

## Background

Although there is growing evidence that tobacco smoke exposure has harmful effects on child health, 40% of children are still exposed to secondhand smoke (SHS) worldwide [1]. Even developing fetuses are exposed to SHS through the umbilical cord blood when mothers are exposed to tobacco smoke [2]. The development and maturation of lung is a complex process from fetal life to adolescence [3]. The developing lung is very sensitive to environmental factors; previous epidemiological and experimental studies revealed a significant suppression of alveolarization, functional residual capacity, and tidal flow volume in children exposed to SHS [4]. Thus, exposure to SHS in fetal life and early postnatal life may have significant effects on the developing lungs including long-lasting structural changes, altered pulmonary function, and an increased risk of asthma and wheeze [3, 5].

Wheeze is a common symptom in young children [6]. In childhood, especially at a young age, distinction between wheezing disorder and asthma is difficult [7]. Although insufficient evidence is available regarding the long-term outcomes of wheeze in preschool children into school age, adolescence, or adulthood [6], early childhood wheeze may be a predictor for atopy, reduced lung function, and diminished airway responsiveness in school age [8], increased risk of asthma in late childhood, and subsequent respiratory disease including asthma in adulthood [9].

A meta-analysis of prospective studies in 2012 revealed that maternal active smoking during pregnancy increases the risk of developing wheeze in children aged 0 to 18 years [10]. Recently, a pooled analysis of 15 European birth cohorts demonstrated that children exposed to prenatal tobacco smoking and both pre- and postnatal tobacco smoking were more likely to develop wheeze up to the age of two years compared with unexposed children [11]. The available evidence regarding the independent and additive effects of both pre- and postnatal smoking exposure on the risk of wheeze in children is limited, however, particularly in Asian populations. Here, to assess the effects of prenatal and postnatal smoking exposure on the risk of wheeze, we investigated the effect of maternal active smoking during pregnancy and postnatal living with smokers on the development of wheeze in children, using data from fetal life to two years of age in the Kyushu Okinawa Maternal and Child Health Study (KOMCHS).

## Methods

### Study population

The KOMCHS is an ongoing prospective birth cohort study that investigates risk and preventive factors for maternal and child health problems. The background and general procedure of the KOMCHS have been described previously [12]. In brief, the KOMCHS requested that pregnant women complete a baseline survey, which was followed by several post-natal surveys. Eligible subjects were those women who became pregnant in one of seven prefectures on Kyushu Island in southern Japan or Okinawa Prefecture between April 2007 and March 2008. At 423 obstetric hospitals in the abovementioned seven prefectures, a set of leaflets explaining the KOMCHS, an application form to participate in the study, and a self-addressed and stamped return envelope were distributed to pregnant women, insofar as this was possible. Pregnant women who intended to participate in the KOMCHS returned the application form to the data management center. In the end, a total of 1757 pregnant women between the 5th and 39th week of pregnancy gave their written fully informed consent to participate in the KOMCHS and completed the baseline survey. Of the 1757 women, 1590, 1527, 1430, and 1362 mother-child pairs participated in the second (after delivery), third (approximately four months postpartum), fourth (approximately 12 months postpartum), and fifth (approximately 24 months postpartum) surveys, respectively. Excluded were five pairs who did not participate in the fifth survey at 23 to 29 months postpartum and three pairs because of missing data on the factors under study, leaving data on 1354 pairs available for analysis. The ethics committees of the Faculty of Medicine, Fukuoka University and Ehime University Graduate School of Medicine approved the KOMCHS.

### Measurements

Each survey consisted of a self-administered questionnaire about lifestyle and other social conditions including smoking, eating, and medical history for mothers at the baseline survey and for mothers and children during the follow-up surveys. Participants filled out the questionnaires and then mailed them to the data management center at the time of each survey. Research technicians completed missing or illogical data by telephone interview.

The baseline survey questionnaire elicited information on region of residence, number of children, maternal and paternal education levels, household income, and maternal and paternal history of asthma, atopic eczema, and allergic rhinitis. Maternal or paternal history of asthma, atopic eczema, or allergic rhinitis was defined as positive if the respective parent had been diagnosed by a physician as having any of these allergic diseases.

The second survey included questions about the baby’s sex, birth weight, and maternal active smoking during pregnancy. Maternal smoking status during pregnancy was assessed with the following questions in the first, second, and third trimesters: “Did you smoke almost every day?” (no smoking/quitting smoking/smoking). The third and fourth surveys asked about the smoking habits of the adult household members and breastfeeding duration. Postnatal smoking exposure was assessed with the following questions: “Did your child live with a smoker after the second survey?” (in the third survey) (yes/no) and “Did your child live with a smoker after the third survey?” (in the fourth survey) (yes/no). Postnatal living with at least one household smoker was defined as positive if the child had lived with at least one smoker at the third survey or at the fourth survey. Breastfeeding duration was defined as the length of the period during which infants received breast milk, regardless of exclusivity. The questionnaire in the fifth survey included question on allergic disorders. Wheeze during the previous year was defined as a positive response to the following question, based on the International Study of Asthma and Allergies in Childhood (ISAAC) criteria: ‘Has your child had wheezing or whistling in the chest in the last 12 months?’

### Statistical analysis

Maternal smoking during pregnancy was classified into four categories (none, first trimester only, second and/or third trimesters but not throughout pregnancy, and throughout pregnancy), and postnatal living with at least one household smoker into two (yes and no). Region of residence at baseline, number of children at baseline, maternal and paternal education levels, household income, maternal and paternal history of asthma, atopic eczema, and allergic rhinitis, infant’s birth weight, infant’s sex, and breastfeeding duration were a priori selected as potential confounding factors.

Multiple logistic regression analysis was performed to estimate adjusted odds ratios (ORs) and 95% confidence intervals (CIs) of wheeze relative to categories of prenatal and postnatal smoking exposure. We also performed an analysis to study the additive effect of prenatal and postnatal smoking exposure. All statistical analyses were performed using the SAS software package version 9.4 (SAS Institute, Inc., Cary, NC, USA).

## Results

At 23 to 29 months of age, 373 of the 1354 children (27.6%) fulfilled the ISAAC criteria for wheeze. About 8% of children were exposed to maternal active smoking at any time during pregnancy, and 44% of children had lived postnatally with at least one household smoker during the first year of life. The characteristics of study subjects are given in Table 1. About 40% of the subjects had no siblings. The mean birth weight of our study subjects was 3002 g. Approximately 89% of the children were breastfed for six months or longer. The numbers of children whose mothers smoked during pregnancy and who postnatally lived with at least one household smoker were 111 and 601, respectively. Children with prenatal or postnatal smoking exposure are more likely to have parents with low educational levels and to live in a family with a low incomes than are those with no exposure to smoking (Additional file 1: Tables S1 and S2).

**Table 1:** Distribution of selected characteristics in 1354 parent–child pairs, Kyushu Okinawa Maternal and Child Health Study, Japan

Variable	No. (%) or mean ± SD
Baseline characteristics	
Region of residence	
Fukuoka Prefecture	783 (57.8)
Other than Fukuoka Prefecture in Kyushu	442 (32.6)
Okinawa Prefecture	129 (9.5)
No. of living children already born to same mother	
0	541 (40.0)
1	543 (40.1)
≥ 2	270 (19.9)
Maternal education, years	
< 13	293 (21.6)
13 − 14	455 (33.6)
≥ 15	606 (44.8)
Paternal education, years	
< 13	409 (30.2)
13 − 14	198 (14.6)
≥ 15	747 (55.2)
Household income, yen/year	
< 4,000,000	454 (33.5)
4,000,000 − 5,999,999	497 (36.7)
≥ 6,000,000	403 (29.8)
Maternal history of asthma	179 (13.2)
Maternal history of atopic eczema	246 (18.2)
Maternal history of allergic rhinitis	564 (41.7)
Paternal history of asthma	147 (10.9)
Paternal history of atopic eczema	133 (9.8)
Paternal history of allergic rhinitis	374 (27.6)
Characteristics at follow-up surveys	
Male sex	646 (47.7)
Birth weight, mean ± SD, g	3002.3 ± 395.5
Breastfeeding duration, mo	
< 6	156 (11.5)
≥ 6	1198 (88.5)

Table 2 shows crude and adjusted ORs and their 95% CIs for wheeze in relation to prenatal and postnatal smoking exposure. Compared with no maternal smoking during pregnancy, maternal smoking throughout pregnancy was significantly associated with an increased risk of wheeze in children; adjusted OR was 2.24 (95% CI: 1.14–4.36). Neither maternal smoking in the first trimester only nor maternal smoking in the second and/or third trimesters but not throughout pregnancy was associated with the risk of wheeze. No association was observed between postnatally living with at least one household smoker during the first year of life and the risk of wheeze.

**Table 2:** ORs and 95% CIs for wheeze according to maternal smoking during pregnancy and postnatal SHS exposure at home, Kyushu Okinawa Maternal and Child Health Study, Japan

	Wheeze in a previous year		
	Risk (%)	Crude OR (95% CI)	Adjusted OR^a^ (95% CI)
Maternal smoking status			
None	330/1243 (26.6%)	1.00	1.00
First trimester only	21/60 (35.0%)	1.49 (0.85–2.54)	1.56 (0.87–2.74)
Second and/or third trimesters but not throughout	3/11 (27.3%)	1.04 (0.23–3.61)	1.23 (0.26–4.44)
Throughout	19/40 (47.5%)	2.50 (1.32–4.72)	2.24 (1.14–4.36)
Postnatal living with at least one household smoker			
No	195/753 (25.9%)	1.00	1.00
Yes	178/601 (29.6%)	1.20 (0.95–1.53)	1.17 (0.91–1.51)

^a^Adjustment for region of residence at baseline, number of children at baseline, maternal and paternal education levels, household income, maternal and paternal history of asthma, atopic eczema, and allergic rhinitis, infant’s birth weight, infant’s sex, and breastfeeding duration

*Abbreviations: CI* confidence interval, *OR* odds ratio, *SHS* secondhand smoke

In an analysis of the additive effect of prenatal maternal smoking and postnatal living with at least one household smoker on wheeze, the children were classified into four mutually exclusive categories (Table 3). Compared with children not exposed to maternal smoking during pregnancy and not postnatally living with at least one household smoker, those who lived postnatally with at least one household smoker in addition to being exposed to maternal smoking during pregnancy, regardless of the trimester in which exposure occurred, had higher odds of wheeze; adjusted OR was 2.04 (95% CI: 1.25–3.31). No association was observed between exposure to maternal smoking during pregnancy only or postnatal living with at least one household smoker only and the risk of wheeze.

**Table 3:** Additive effect of maternal smoking during pregnancy and postnatal SHS exposure at home, Kyushu Okinawa Maternal and Child Health Study, Japan

		Wheeze in a previous year		
Maternal smoking during pregnancy	Postnatal living with at least one household smoker	Risk (%)	Crude OR (95% CI)	Adjusted OR^a^ (95% CI)
No	No	190/732 (26.0%)	1.00	1.00
Yes	No	5/21 (23.8%)	0.89 (0.29–2.31)	0.96 (0.30–2.59)
No	Yes	140/511 (27.4%)	1.08 (0.83–1.39)	1.06 (0.81–1.39)
Yes	Yes	38/90 (42.2%)	2.09 (1.32–3.26)	2.04 (1.25–3.31)

^a^Adjustment for region of residence at baseline, number of children at baseline, maternal and paternal education levels, household income, maternal and paternal history of asthma, atopic eczema, and allergic rhinitis, infant’s birth weight, infant’s sex, and breastfeeding duration

*Abbreviations: CI* confidence interval, *OR* odds ratio, *SHS* secondhand smoke

## Discussion

In the present pregnancy birth cohort study, we found that maternal smoking throughout pregnancy, but not during the first trimester only or the second and/or third trimesters but not throughout, increased the risk of wheeze in children. No association was observed between postnatal living with at least one household smoker in the first year of life and wheeze development.

A population-based prospective cohort study in the Netherlands demonstrated that continued maternal smoking during pregnancy was associated with an increased risk of wheeze until age six, whereas there was no statistically significant association between maternal smoking in the first trimester only and the risk of wheeze [13, 14]. The results for prenatal smoking exposure in our study are partially consistent with those of previous studies. Most previous studies on the association between maternal smoking during pregnancy and the risk of wheeze have not been able to assess the effect of maternal smoking exposure in different stages of pregnancy. In a meta-analysis in 2012, maternal smoking during pregnancy was associated with a 70% greater risk of wheeze in children aged two and younger, although exposure in different stages of pregnancy was not taken into account [10]. Our results showing no association between postnatal living with at least one household smoker and the risk of wheeze are consistent with those of some previous studies that likewise found no association between postnatal smoking exposure and wheeze in childhood [15–17], but are at variance with those of other studies that found a positive association [18–20]. Thus the evidence regarding the association between postnatal smoking exposure and risk of wheeze in children remains inconclusive.

Our results concerning the independent and joint effects of both pre- and postnatal smoking exposure are in agreement with those of a Danish pre-birth cohort study in which both pre- and postnatal smoking exposure increased the risk of wheeze in children aged 14 to 18 years, while there was no association between prenatal smoking exposure only or postnatal SHS exposure only and the risk of wheeze [21]. On the other hand, our previous cross-sectional study showed that postnatal smoking exposure only, but not prenatal smoking exposure only or both pre- and postnatal smoking exposure, was positively associated with the prevalence of wheeze among children aged three years [22]. According to a pooled analysis of eight European birth cohort studies, children exposed to maternal smoking during pregnancy only and maternal smoking during pregnancy as well as in the first year of life had an increased risk of wheeze at 4 to 6 years of age, whereas no significant association was observed for children exposed to maternal smoking during the first year of life only [23]. In the present study, no association was observed between maternal smoking during pregnancy only and the risk of wheeze in an analysis of the additive effect. This might be ascribed to the small number of children whose mothers smoked during pregnancy and who did not live with at least one household smoker during the first year of life (*n* = 21). Alternatively, the periods of the trimester of pregnancy in which mothers smoked was not taken into account. Thus, the crude classification of maternal smoking during pregnancy might explain our observed lack of an association.

Tobacco smoke contains 4000 chemicals, at least 250 of which are toxic to the human body [24]. The toxic components of tobacco smoke may also reach the fetus by crossing the placenta [25]. The developing lung is highly susceptible to environmental exposure. Because the process of lung development continues from embryogenesis into postnatal life, exposure to tobacco smoke during any of these critical periods might affect the structure and function of the respiratory system, leading to impaired lung function and an increased risk of respiratory disorders, particularly the occurrence of respiratory infections and asthma/allergy symptoms [25, 26].

One strength of our study was its prospective design, in which subjects were followed from the fetal period onward, minimizing the effect of recall bias. In addition, a number of potential confounding factors were controlled for in the analysis, although residual confounding or confounding by other potentially important factors still remains a possibility.

This study has some limitations. First, of the 1757 participants at baseline, only 1354 (77.1%) children were evaluated in the present study. Nonparticipation in follow-up surveys or the exclusion of subjects due to incomplete information could have led to biased effect estimates. Compared with children who were excluded from the current study (*n* = 403), study subjects were more likely to live in Fukuoka Prefecture, to live in families with relatively high incomes and to have parents with relatively high educational levels, and their mothers were less likely to have smoked during pregnancy. There were no material differences between included and excluded children regarding distribution of the number of children at baseline or parental history of asthma, atopic eczema and allergic rhinitis. Moreover, selection bias might have occurred at the baseline survey. The participation rate could not be calculated because the exact number of eligible pregnant women who were provided with a set of leaflets explaining the KOMCHS, an application form, and a self-addressed and stamped return envelope by the 423 collaborating obstetric hospitals is not available. Our subjects were probably not a representative sample of the Japanese population. In fact, the educational levels of the parents of the current study population were higher than those of the general population [27]. Thus our study subjects were more educated and may therefore have had a greater awareness about health than the general population.

Second, this study is subject to the limitations inherent in questionnaire-based studies. As the outcome variable was based on maternal reporting, misclassification of outcome could result from maternal recall errors. Data on smoking exposure were self-reported and were not confirmed by objective measurements. We cannot exclude the possibility of reporting bias due to underreporting by the participants, leading to misclassification of exposure resulting in underestimation of the true associations. Although data on maternal smoking during pregnancy were collected prior to the development of wheeze in children, such data were retrospectively collected by the second questionnaire after delivery. Thus, the possibility of recall bias should be taken into account. In the present study, information on the number of cigarettes smoked by mothers or household members was not available. Therefore, we cannot assess the dose–response relationship between prenatal and postnatal smoking exposure and the risk of wheeze in children.

Third, although the definition of wheeze was based on the ISAAC questions, misclassification may have occurred because the validity of the ISAAC questions is uncertain in infants. No objective measures of atopy or asthma were available in the present study.

Finally, we were not able to take into consideration postnatal exposure to SHS in settings other than the home. Postnatal exposure to SHS may therefore be underestimated.

## Conclusions

The present pregnancy birth cohort study demonstrated that maternal smoking throughout pregnancy increases the risk of childhood wheeze. We also found a positive additive effect of maternal smoking during pregnancy and postnatal living with at least one household smoker on the risk of wheeze in children. In order to clarify the effects of exposure at various stages during pregnancy and the independent and additive effects of prenatal and postnatal smoking exposure on the risk of wheeze, additional evidence is needed.

## Additional file

Additional file 1: Table S1. Distribution of selected characteristics in 1354 parent-child pairs according to prenatal smoking exposure status, Kyushu Okinawa Maternal and Child Health Study, Japan. **Table S2.** Distribution of selected characteristics in 1354 parent-child pairs according to postnatal smoking exposure status, Kyushu Okinawa Maternal and Child Health Study, Japan. (DOCX 20 kb)

## Abbreviations

CI: Confidence interval
ISAAC: International study of asthma and allergies in childhood
KOMCHS: Kyushu Okinawa maternal and child health study
OR: Odds ratio
SHS: Secondhand smoke
