# Supplementary material for: Secondhand smoke exposure and risk of wheeze in early childhood: a prospective pregnancy birth cohort study
Source: Tob Induc Dis. 2017 Jul 18;15:30. doi: 10.1186/s12971-017-0138-7 (PMC5516318; doi:10.1186/s12971-017-0138-7)
Supplement: Additional file 1: Table S1. — Distribution of selected characteristics in 1354 parent-child pairs according to prenatal smoking exposure status, Kyushu Okinawa Maternal and Child Health Study, Japan. Table S2. Distribution of selected characteristics in 1354 parent-child pairs according to postnatal smoking exposure status, Kyushu Okinawa Maternal and Child Health Study, Japan. (DOCX 20 kb) [file 12971_2017_138_MOESM1_ESM.docx]

**Supplemental Table 1** Distribution of selected characteristics in 1354 parent-child pairs according to prenatal smoking exposure status, Kyushu Okinawa Maternal and Child Health Study, Japan

| Variable | Maternal smoking during pregnancy | | *P* value |
| --- | --- | --- | --- |
|  | Yes (n = 111) | No (n =1243) |  |
|  | No. (%) or mean ± SD | No. (%) or mean ± SD |  |
| **Baseline characteristics** |  |  |  |
| Region of residence |  |  |  |
| Fukuoka Prefecture | 62 (55.9) | 721 (58.0) | 0.005 |
| Other than Fukuoka Prefecture in Kyushu | 29 (26.1) | 413 (33.2) |  |
| Okinawa Prefecture | 20 (18.0) | 109 (8.8) |  |
| No. of living children already born to same mother |  |  |  |
| 0 | 55 (49.6) | 486 (39.1) | 0.03 |
| 1 | 32 (28.8) | 511 (41.1) |  |
| ≥ 2 | 24 (21.6) | 246 (19.8) |  |
| Maternal education, years |  |  |  |
| < 13 | 48 (43.2) | 245 (19.7) | < 0.0001 |
| 13−14 | 38 (34.2) | 417 (33.6) |  |
| ≥ 15 | 25 (22.5) | 581 (46.7) |  |
| Paternal education, years |  |  |  |
| < 13 | 59 (53.2) | 350 (28.2) | < 0.0001 |
| 13−14 | 19 (17.1) | 179 (14.4) |  |
| ≥ 15 | 33 (29.7) | 714 (57.4) |  |
| Household income, yen/year |  |  |  |
| < 4,000,000 | 57 (51.4) | 397 (31.9) | < 0.0001 |
| 4,000,000−5,999,999 | 36 (32.4) | 461 (37.1) |  |
| ≥ 6,000,000 | 18 (16.2) | 385 (31.0) |  |
| Maternal history of asthma | 24 (21.6) | 155 (12.5) | 0.006 |
| Maternal history of atopic eczema | 18 (16.2) | 228 (18.3) | 0.58 |
| Maternal history of allergic rhinitis | 46 (41.4) | 518 (41.7) | 0.96 |
| Paternal history of asthma | 12 (10.8) | 135 (10.9) | 0.99 |
| Paternal history of atopic eczema | 6 (5.4) | 127 (10.2) | 0.10 |
| Paternal history of allergic rhinitis | 28 (25.2) | 346 (27.8) | 0.56 |
| **Characteristics at follow-up surveys** |  |  |  |
| Male sex | 43 (38.7) | 603 (48.5) | 0.05 |
| Birth weight, mean ± SD, g | 2916.8 ± 417.8 | 3009.9 ± 392.7 | 0.02 |
| Breastfeeding duration, mo |  |  |  |
| < 6 | 23 (20.7) | 133 (10.7) | 0.002 |
| ≥ 6 | 88 (79.3) | 1110 (89.3) |  |

**Supplemental Table 2** Distribution of selected characteristics in 1354 parent-child pairs according to postnatal smoking exposure status, Kyushu Okinawa Maternal and Child Health Study, Japan

| Variable | Postnatal living with at least one household smoker | | *P* value |
| --- | --- | --- | --- |
|  | Yes (n = 601) | No (n =753) |  |
|  | No. (%) or mean ± SD | No. (%) or mean ± SD |  |
| **Baseline characteristics** |  |  |  |
| Region of residence |  |  |  |
| Fukuoka Prefecture | 344 (57.2) | 439 (58.3) | 0.25 |
| Other than Fukuoka Prefecture in Kyushu | 207 (34.4) | 235 (31.2) |  |
| Okinawa Prefecture | 50 (8.3) | 79 (10.5) |  |
| No. of living children already born to same mother |  |  |  |
| 0 | 221 (36.8) | 320 (42.5) | 0.07 |
| 1 | 248 (41.3) | 295 (39.2) |  |
| ≥ 2 | 132 (22.0) | 138 (18.3) |  |
| Maternal education, years |  |  |  |
| < 13 | 172 (28.6) | 121 (16.1) | < 0.0001 |
| 13−14 | 211 (35.1) | 244 (32.4) |  |
| ≥ 15 | 218 (36.3) | 388 (51.5) |  |
| Paternal education, years |  |  |  |
| < 13 | 226 (37.6) | 183 (24.3) | < 0.0001 |
| 13−14 | 96 (16.0) | 102 (13.6) |  |
| ≥ 15 | 279 (46.4) | 468 (62.2) |  |
| Household income, yen/year |  |  |  |
| < 4,000,000 | 251 (41.8) | 203 (27.0) | < 0.0001 |
| 4,000,000−5,999,999 | 217 (36.1) | 280 (37.2) |  |
| ≥ 6,000,000 | 133 (22.1) | 270 (35.9) |  |
| Maternal history of asthma | 83 (13.8) | 96 (12.8) | 0.57 |
| Maternal history of atopic eczema | 111 (18.5) | 135 (17.9) | 0.80 |
| Maternal history of allergic rhinitis | 253 (42.1) | 311 (41.3) | 0.77 |
| Paternal history of asthma | 60 (10.0) | 87 (11.6) | 0.36 |
| Paternal history of atopic eczema | 46 (7.7) | 87 (11.6) | 0.02 |
| Paternal history of allergic rhinitis | 129 (21.5) | 245 (32.5) | < 0.0001 |
| **Characteristics at follow-up surveys** |  |  |  |
| Male sex | 298 (49.6) | 348 (46.2) | 0.22 |
| Birth weight, mean ± SD, g | 3002.3 ± 420.6 | 3002.3 ± 374.5 | 1.00 |
| Breastfeeding duration, mo |  |  |  |
| < 6 | 90 (15.0) | 66 (8.8) | 0.0004 |
| ≥ 6 | 511 (85.0) | 687 (91.2) |  |
